# The Use of Adipose Stem Cells in Cranial Facial Surgery

**DOI:** 10.1007/s12015-014-9522-3

**Published:** 2014-06-10

**Authors:** Michelle Griffin, Deepak M. Kalaskar, Peter E. Butler, Alexander M. Seifalian

**Affiliations:** 1 UCL Centre for Nanotechnology and Regenerative Medicine, Division of Surgery & Interventional Science, University College London, London, United Kingdom; 2 Royal Free London NHS, Foundation Trust Hospital, London, United Kingdom

**Keywords:** Cranial facial surgery, Adipose derived stem cells, Osteogenesis, Chondrogenesis, Adipogenesis

## Abstract

Craniofacial malformations, have devastating psychosocial implications for many adults and children and causes huge socioeconomic burden. Currently craniofacial defects require soft tissue transfer, bone grafting techniques or difficult procedures such as microvascular free flaps. Such tissues are often limited in quantity, their harvest causes secondary large donor site defects and they lack the capability to fully restore previous form and function. Stem cell technology is being utilised for various tissue and organs of the body and consequently surgeons are eager to transfer these principles for craniofacial surgery. Adipose derived stem cells (ADSCs) are an exciting stem cell source for craniofacial surgeons due to their easy and painless isolation, relatively large abundance and familiarity with the harvesting procedure. ADSCs also have multiple desirable properties including adipogenic, osteogenic and chondrogenic potential, enhancement of angiogenesis and immunodulatory function. Due to these advantageous characteristics, ASDCs have been explored to repair craniofacial bone, soft tissue and cartilage. The desirable characteristics of ADSCs for craniofacial surgical applications will be explained. We report the experimental and clinical studies that have explored the use of ADSCs for bone, cartilage and soft tissue craniofacial defects. We conclude by establishing the key questions that are preventing the clinical application of ADSCs for craniofacial surgery.

## Introduction

As the field of regenerative medicine continues to grow, there is a need for a reliable and continuous source of stem cells that can be easily obtained. Mesenchymal stem cells can be isolated from various tissue sources in adults [1]. For many years, bone marrow derived stem cells (BMDSCs) have been the focus of tissue engineering research strategies. However, current research interest is looking towards developing adipose derived stem cells (ADSCs), which are isolated directly from either fat excision or liposuction during plastic surgical procedures. ADSCs share many properties as those of BMDSCs with similar potential to differentiate into bone, cartilage, muscle and fat [2]. However, with easier isolation and better availability ADSCs have sparked great clinical and research interest. There have already been several clinical reports of the successful application of ADSCs including soft tissue augmentation [3], wound healing [4] and Crohn’s disease [5].

Craniofacial surgery is particularly suited to the implementation of ADSCs due to the huge demand for reconstruction of several tissue types. Firstly, there is a huge demand for soft tissue reconstruction including (1) reconstruction of surgically or traumatically created facial tissue voids (2) to restore bulk of aging tissue in order to correct soft tissue folds (3) to augment or create soft tissue for cosmetic enhancement and lastly (4) to create soft tissue contours for patients with congenital soft tissue deficiencies. Secondly, there is strong clinical need to generate bone for craniofacial osseous defects due to congenital diseases, trauma and surgically created bony defects following cancer resections. The paediatric population represents a large clinical need for tissue-engineered bone including cleft palate, Down syndrome, Treacher Collins syndrome and Apert and Crouzon syndromes. Autogenous bone grafts harvested from the iliac bone are considered to be the gold standard to treat bone defects but this causes huge donor site morbidity, pain and has limited availability. Calvarium defects represent a particular reconstructive challenge as above the age of 2 years it does not regenerate on its own [6]. Lastly cartilage is often required following trauma, inflammatory conditions or cancer resections of the nasal area or in paediatric patients for auricular reconstruction due to microtia and anotia.

**Fig. 1:**
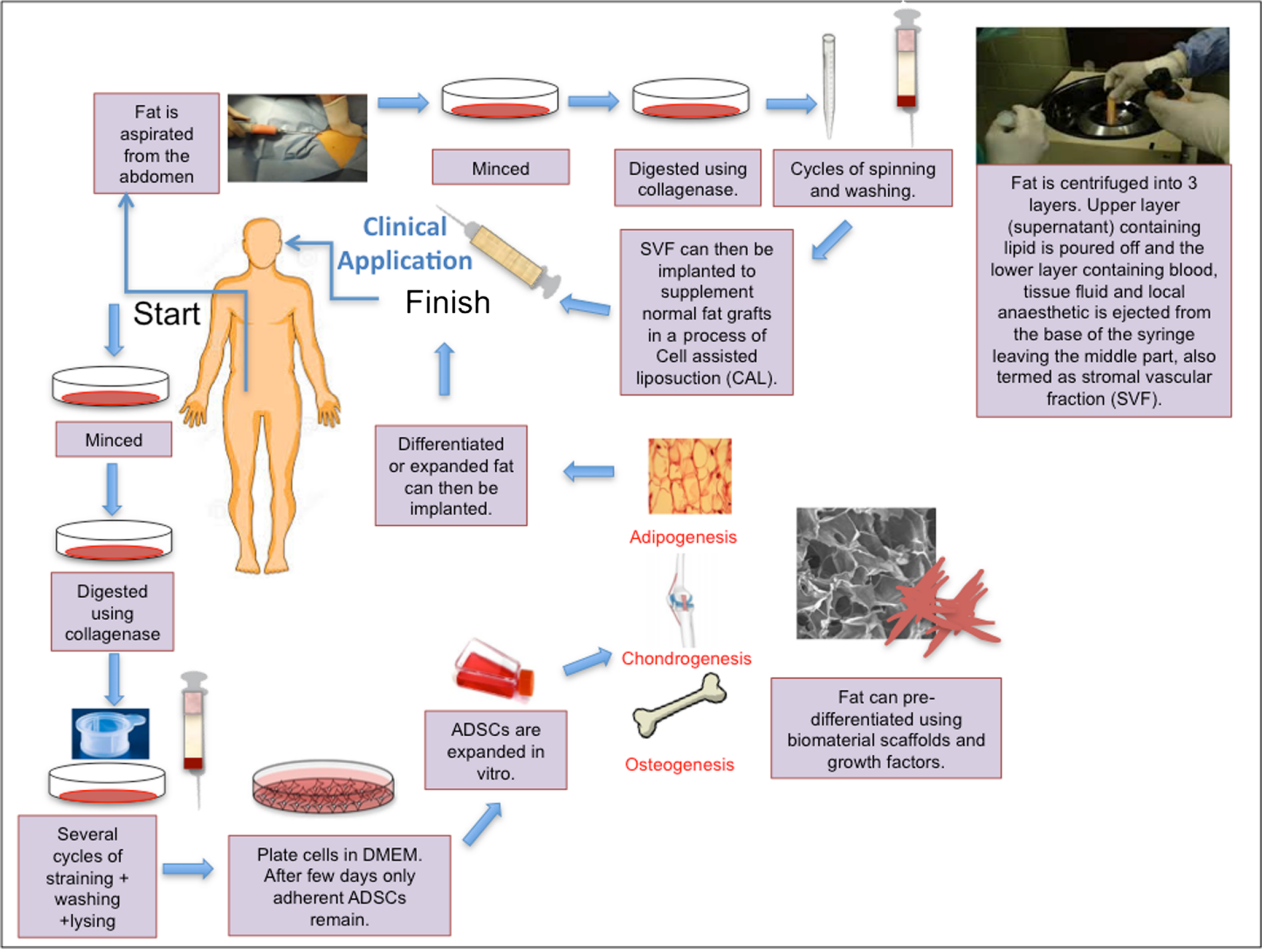
Schematic drawing illustrating how adipose stem cells can be utilised for craniofacial surgery. Adipose derived stem cells (ADSCs) can supplement fat grafts for improved adipogenesis in a process called cell assisted liposuction (CAL). Furthermore, ADSCs can be expanded in vitro under Good Manufacturing Practice and Good Laboratory Practice before clinical use. Lastly ADSCs inserted on biomaterials with/without growth factors is being explored to enhance their differentiation towards certain cell lineages. ADSCs; Adipose derived stem cells. DMEM; Dulbecco Modified Eagle Medium

The “gold standard” to address these defects currently involves using autologous material in the form of soft tissue transfer, bone or cartilage grafting and complicated free flaps and microvascular anastomosis [7, 8]. Such techniques create secondary donor defects with associated risks and complications and are limited in their availability. Various alloplastic materials are available to replace autologous tissue including silicone, medpor, titanium but all have shown mechanical failure, extrusion, infection and limited capability to recreate previous form [9–11]. It has been suggested that ADSC strategies can overcome the necessary donor site morbidity, limited availability and failure of autologous grafts and extrusion and infection of alloplastic grafts [12, 13]. Various techniques for using ADSCs for clinical applications are shown in Fig. 1.

This review aims to describe the desirable properties of ADSCs currently in practice. Further, we evaluate the evidence that uses ADSCs for bone, cartilage and adipose engineering currently in practice for craniofacial reconstruction surgery and lastly describe the possibilities and expected contribution of ADSCs in the future of craniofacial surgery.

## What are Adipose Stem Cells?

Adipose tissue is one of the largest tissues in the body, acting as an important energy and endocrine reservoir [14]. Adipose tissue is mainly composed of adipocytes (fat cells), accounting for more than 90 % of the tissue volume, being arranged in lobules [15]. Adipose tissue is a very vascular tissue with all of the adipocytes in contact with the surrounding capillaries [14]. In addition to the adipocytes, adipose tissue consists of pericytes, fibroblasts, macrophages, vascular endothelial cells and an extracellular matrix [15]. ADSCs were first identified in 2001 by Zuck et al. as a population of fibroblast cells capable of differentiating into adipogenic, chondrogenic, myogenic and osteogenic cells in the presence of specific induction factors [2]. Since this time many groups have tried to optimise the isolation and expansion of ADSCs [16–27]. ADSC are commonly extracted from adipose tissue in a multi-step wise procedure. Adipose tissue is first obtained through liposuction, from a variety of sites including the upper arm, medial thigh, trochanteric, and superficial deep abdominal depots [21]. The most commonly used ADSC isolation technique is that described by Coleman. Processing the lipoaspirate via the Coleman method involves centrifugation of lipoaspirate at 3,000 rpm for 3 min in 10 ml syringes. The processed harvested fat is then separated into three layers. The upper layer (supranatant) containing lipid is poured off and the lower layer containing blood, tissue fluid and local anaesthetic is ejected from the base of the syringe leaving the middle part containing stromal cells, vascular endothelial and mural cells, termed the stromal vascular fraction (SVF). To isolate ADSCs from the adipose tissue, it is common to digest the adipose tissue using collagenase after mincing and cutting of the tissue. After neutralisation with Dulbecco Modified Eagle Medium (DMEM) containing foetal bovine serum (FBS) and centrifugation an ADSC-rich pellet is formed. This is then cultured and expanded in media. Following a period of several days or hours the non-adherent cells are then removed with the remaining cells being ADSCs [27] (Fig. 1).

### Useful Properties of Adipose Stem Cells

Human adipose tissue offers several advantages as a stem cell source (Table 1). With the widespread obesity in current populations, most adults have abundant adipose tissue [28]. In addition, the technique described in Fig. 1 for adipose harvesting is relatively less painful compared to the bone marrow aspiration with decreased donor site morbidity and 10–100 times greater frequency of stromal cells per unit volume [29]. ADSCs are attractive options for tissue regeneration due to their angiogenic, wound healing and immunodulatory properties [30–45].

**Table 1:** Properties of adipose derived stem cells (ADSCs) that are valuable for cranial facial surgery

Property	Study	Mechanism	Outcome	Reference
Enhance angiogenesis	Mouse ADSCs to mouse hind limb	Production of Cytokines (SDF-1, VEGF)	At 3 weeks, ADSC group had greater perfusion index and a higher capillary density compared to controls.	30
	Mouse SVF to mouse hind limb	EC and SMC differentiation	SVFs significantly increased vascular collateral development and capillary density of ischemic muscle.	31
	Mouse ADSC to mouse hind limb	Production of Cytokines (VEGF, HGF)	At 4 weeks after transplantation of ADSCs into the ischemic mouse hindlimb, the angiogenic scores were improved in the ADSC-treated group.	32
	Human SVF to mouse hind limb	EC differentiation	Cultured human SVF cells differentiate into endothelial cells, incorporate into vessels, and promote both post-ischemic neovascularisation in nude mice.	33
	ADSCS to hindlimb of nude mice with FGF-2	Production of Cytokine (FGF-2, VEGF and HGF)	ADSCs stimulated tube formation in an in vitro tube formation assay.	34
Enhance wound healing	Rat diabetic skin graft model	Increase of capillary density, collagen intensity, VEGF, and TGF-β3 expression	The gross and histological results showed increased survival, angiogenesis, and epithelialisation in ADSCs seeded full thickness skin grafts.	35
	Rat cutaneous skin wound	Production of cytokines (epidermal growth factor and vascular endothelial growth factor)	ADSCs enhanced the cell proliferation and neovascularisation of the regenerated skin.	36
	Rat ulcer model	Promote new blood vessel formation	The wound size after ADSCs treatment for 3 weeks was significantly smaller compared to control (*p* < 0.01).	37
	Murine full thickness wound defect	Down-modulate TNF-α-dependent inflammation, increase anti-inflammatory macrophage numbers, and induce TGF-β1-dependent angiogenesis, myofibroblast differentiation and granulation tissue formation	ADSCs delivered to murine wounds accelerated wound healing.	38
	Full thickness rat wound defect	Enhanced total vessel formation after 3 weeks.	The ADSCs group showed smaller injury areas at all time points except day 21 and enhanced wound healing compared to the single layer ADSCs sheet at day 7, 10 and 14.	39
Anti-inflammatory actions	Mouse with SLE	Increasing levels of anti-inflammatory cytokines	ADSCs group showed higher survival rate with improvement of histologic and serologic abnormalities, immunologic function and decreased incidence of proteinuria.	40
	Murine model of arthritis	Decreased inflammatory cytokines and autoimmune TH1 cells	Thickening of the synovial lining, formation of enthesophytes associated with medial collateral ligaments and cruciate ligaments were significantly inhibited on day 42 after ADSC treatment, by 31 %, 89 %, and 44 %, respectively.	41
Suppression of alloreactive T cells	Co-culture of canine ADSCs with leukocyte	Paracrine cytokine production of TGF- β, HGF, prostaglandin E2 (PGE2), and indoleamine-2, 3-dioxygenase (IDO)	Leukocyte proliferation induced by mitogens was suppressed when co-cultured with irradiated ADSCs.	42
	Co-culture of human ADSCs and dendritic and T lymphocytes	Paracrine secretion of PGE2	ADSCs inhibited the maturation of myeloid dentritic cells and plasmocytoid-dentritic cells.	43
	Mouse ADSCs prevented graft versus host disease in mice transplanted with haploid identical hematopoietic grafts	1. Inhibit the production of inflammatory cytokines (TNF-α, IFN- γ, and IL-12) of T cells so not to induce proliferation of allogeneic T cells	Infusion of ADSCs in mice transplanted with haploidentical haematopoietic grafts controlled the lethal GVHD that occurred in control recipient mice.	44
		2. Suppress the proliferation of T cells induced either by mitogens or allogeneic cells		

Keys: *EC* endothelial cell; *ADSCs* adipose derived stem cell, *SMC* smooth muscle cell, *SDF*-*1* stromal derived factor-1, *HGF* hepatocyte growth factor, *FGF*-*2* fibroblast growth factor-2, *TGF*- *β1* transforming growth factor-B1, *IL*-*12 Interleukin 12*, *VEGF* vascular endothelilal growth factor, *TNF*-*α* transforming growth factor-α, *IFN*- *γ* interferon- γ, *SLE* systemic lupus erythematosus

## Current Evidence Supporting the Use of ADSCs for Craniofacial Surgery

ADSCs are an exciting stem cell source for craniofacial surgeons due to the capacity to facilitate angiogenesis, limit apoptosis, provide immunodulatory function and multi-differentiation capacity. Preclinical and clinical trials are already exploring the potential of ADSCs for reconstructive surgery. For craniofacial surgery bone, cartilage, fat tissues are required for various reconstructive applications. This section will discuss each of these requirements in detail.

### Use of ADSCs to Generate Bone

ADSCs can undergo osteogenic differentiation in vitro by exposure to a combination of ascorbate, β-glycerophosphate, various bone morphogenetic proteins (BMPs), dexamethasone, and/or vitamin D3, confirming bone formation using Alizarin Red or Von Kossa staining, usually over a two-week period [45]. Several studies have shown that ADSCs will express multiple markers for osteogenesis in these conditions including cbfa-1, alkaline phosphatase, osteopontin, osteocalcin and collagen I [46, 47].

Repair of large bone defects is a common challenge to craniofacial reconstructive surgeons. The current gold standard of current restoration is to use autologous bone to reconstruct craniofacial defects, which is often insufficient in quantity and causes huge donor site morbidity. Despite alloplastic materials and prosthetic implants including metal and plastics trying to act as alternatives, optimal clinical results are not achieved for cranial restoration. However, by delivering osteogenic induced cells or cells capable of osteogenesis such as ADSCs, bone formation for cranial bone defects could be achieved. Several pre-clinical studies have utilised ADSCs for engineering bone to repair cranial defects and few clinical cases have also been reported.

Calvarial defects are the more frequently used model to test stem cells for tissue engineering. Several rodent animal studies have illustrated the ability of ADSCs to form bone to heal calvarial defects. Human ADSCs isolated from the fat tissue of 3 patients was harvested from the abdominal tissue discarded during reconstructive breast surgery. The ADSCs were then seeded on polylactic glycolic acid, atelocollagen, and hydroxyapatite scaffolds to support osteogenesis in athymic nude rat calvaria [48]. Bone mineral densitometry analysis revealed a 2 to 3-fold increase in mineral density in ADSC-seeded scaffolds and healed the rat calvarial defects [48]. Osteogenically induced ADSCs have been thought to provide better bone formation than unstimulated ADCSs. Di bella et al. highlighted that osteogenically induced ADSCs can promote more bone formation than unstimulated ADSCs in a rabbit model. Osteogenically induced ADSCs seeded on fibronectin treated Poly(lactic acid) (PLA) scaffolds formed significantly more bone than PLA scaffolds without fibronectin and scaffolds with undifferentiated ADSCs (*p* < 0.0005) over 6 weeks [49]. The immunomodulatory functions of allogenic ADSCs has been utilised by healing a cranial critical sized defect without the need of immunosuppressive therapy on a coral scaffold [50].

Few studies, have illustrated that the combination of growth factors and ADSCs can support the healing of calvarial bone defects. Xenograft bone chips, covered with acellular periosteum with ADSC and progenitor stem cells and vascular endothelial growth factor and/or bone morphogenetic protein-2 (BMP-2) showed histological confirmation of bone healing for rat critical calvarial bone defects.[51] Lin et al. showed that BMP-2 transfected ADSCs loaded on alginate showed complete healing of rat calvarial cranial defects of 16 weeks but only partial repair for scaffold alone and non transfected ADSCs [52]. Lin et al. illustrated that osteogenic differentiation by BMP-4 adenovirus of BMDSCs and ADSCs was capable of healing rabbit calvarial defects [53].

Whilst there are numerous reports in rodents, there are only two clinical studies showing the capacity of ADSCs for calvarial bone regeneration. The first was the case report of using ADSCs to reconstruct widespread calvarial defects of a 7-year-old girl following a severe head injury [54]. ADSCs were combined with fibrin glue and bone from the iliac crest to reconstruct the calvarial defect in a single operation. The second study, also reported in Germany, illustrated the reconstruction of large calvarial defects in 4 patients using ADSC seeded in beta-tri-calcium phosphate granules [55].

Several large animal studies have illustrated the potential of ADSCs to healing mandibular defects. The injection of ADSCs into the ramus of the pig mandible showed accelerated bone development after 2 and 4 weeks [56]. Similarly, ADSCs seeded onto collatemp scaffolds (collagen impregnated with gentamycin) showed greater bone formation than scaffold alone in a canine mandibular defect [57]. Two clinical studies have confirmed the used of ADSCs for mandible defects. ADCS have been used to reconstruct the critical size defects of the mandible seeded on a resorbable scaffold combined with BMP-2 in 23 patients [58]. More recently, the same author reported the reconstruction of a 10 cm anterior mandibular ameloblastoma resection defect, using a tissue-engineered construct consisting of β-tricalcium phosphate (β-TCP) granules, recombinant human bone morphogenetic protein-2 (BMP-2), and Good Manufacturing Practice (GMP) level autologous ADSCs [59].

**Fig. 2:**
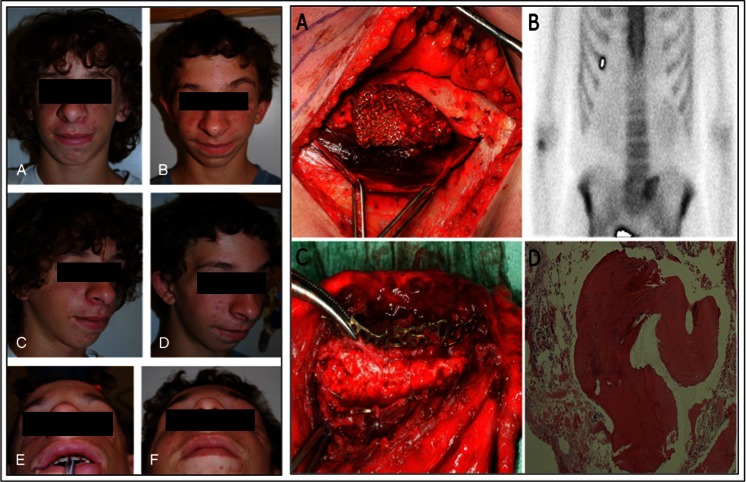
Left: 14-year of boy with Treacher Collins Syndrome treated with human bone allograft, adipose derived stem cells (ADSCs), bone morphogenetic protine-2 (BMP-2) and a periosteal graft to manage his bilateral orbitozygomatic defects. **a**, **c**, **e** Preoperative views. **b**, **d**, **f** postoperative views [60]. Reprinted from Taylor JA. (2010). Bilateral orbitozygomatic reconstruction with tissue-engineered bone. J Craniofac Surg, 21, 1612–4. Right: Maxillary reconstruction following hemimaxillectomy using ADSCs seeded on a titanium cage with beta-tricalcium phosphate (bTCP). (**a**) The titanium cage filled with bTCP and ADSCs, before being inserted into the rectus abdominis muscle pouch [61]. **b** Skeletal scintigraphy of the rectus abdominis muscle was performed which confirmed bone activity [61]. **c** When the rectus abdominis free-flap was raised, and the muscle pouch and titanium cage was opened the tissue engineered bone was clinically confirmed to be rigid. After disconnecting the vessels the flaps was placed in the maxillary defect. [61] **d** A histological section from the tissue-engineered bone showed normal mature bone structures. Reprinted from Mesimäki K, Lindroos B, Törnwall J, Mauno J, Lindqvist C, Kontio R, Miettinen S, Suuronen R. (2009). Novel maxillary reconstruction with ectopic bone formation by GMP adipose stem cells. Int J Oral Maxillofac Surg, 38, 201–9 Copyright (2014), with permission from Elsevier

The use of ADSCs and orbital floor defects has not been thoroughly explored. A single report has also illustrated the success of ADSCs for orbitozygomatic reconstruction [60]. A 14-year of boy with Treacher Collins Syndrome was treated with engineered bone made from a combination of human bone allograft, ADSCs, BMP-2, and periosteal graft to manage his bilateral orbitozygomatic defects (Fig. 2) [60]. Similarly there has been one single case report of using ADSCs for maxillary reconstruction (Fig. 2) [61].

### Use of ADSCs to Generate Cartilage

Cranial cartilage defects involves the auricular and nasal cartilage caused by congenital deformities, cancer resections, inflammatory conditions and trauma. Adult cartilage is avascular, with limited capability of self-restoration due to the matrix having a slow turnover and very low supply of progenitor cells [62]. Therefore, current restoration of cartilage defects involves obtaining autologous cartilage from either costal, auricular or nasal cartilage. However, the limited supply of cartilage, the consequential donor site morbidity, the fact it cannot be easily shaped into the desired shape, are all reasons that researchers are trying to find alternative strategies to create cartilage tissue [62]. Since the work of Cao et al. in 1997, who engineered cartilage using chondrocytes in the shape of an ear in an nude mouse model, many researchers have tried to develop cartilage constructs using tissue engineering principles [63]. In addition to finding the right scaffolds to support chondrogenesis the source of cells to allow for cartilage formation is required.

The expression of chondrogenic markers can be induced in ADSCs in vitro after exposure to a combination of dexamethasone, transforming growth factor (TGF β-1/β-3) and ascorbate confirmed by positive alician blue staining over a three-week period [62]. Several studies have highlighted that 3-dimensional culture (3D) enhanced the chondrogenic differentiation of ADSCs compared to 2-dimensional (2D) culture. The simplest method to achieve this is to culture using a micromass pellet [64]. Alternatively, ADSCs can be seeded on a biomaterial scaffold grown in chondrogenic culture condition. Yoon et al. illustrated that ADSCs showed greater proliferation and differentiation on 3D-hydroxyapatite scaffolds compared to the micromass culture [65].

Several culture medium cocktails have been investigated to induce chondrogenesis of ADSCs and BMDSCs, due to the difficulty to obtain reliable differentiation (Table 2) [66–75]. In addition to the media, several biomaterials have successfully shown to support the differentiation of ASDSCs into cartilage. Natural biomaterials that support chondrogenesis include alginate and fibrin. Autologous ADSCs were isolated and induced with growth medium and placed in a fibrin glue scaffold and into 3 x 4-mm full-thickness chondral defects in rabbits with negative controls. Twelve of 12 (100 %) articular surface defects containing tissue-engineered stem cell constructs healed with hyaline-like cartilage, versus 1 of 12 (8 %) in the control group (p <0.001) [76]. Similarly, alginate disks seeded with ADSCs supported the formation of a cartilaginous like matrix at 12 weeks in nude mice, with increase expression of collagen III, VI and chondroitin sulphate [77].

**Table 2:** Examples of the multiple medium combinations to stimulate chondrogenic differentiation of adipose derived stem cells (Adapted from 66) Source of tissue were from human except *from rabbit

Year and Ref	Differentiation Protocol	Outcome
2001 [2]	DMEM, FBS, insulin, ascorbate 2-phosphate	Processed lipoaspirate cells differentiate in vitro into a chondrogenic lineage using specific induction factors.
2002 [67]	DMEM, FBS, ITS, ascorbate 2-phosphate, dexamethasone, TGFβ-1, sodium pyruvate	ADSCs abundantly synthesized cartilage matrix molecules including collagen type II, VI, and chondroitin 4-sulfate.
2003 [68]	DMEM, BSA, ITS, ascorbate 2-phosphate, sodium pyruvate, TGFβ-1, dexamethasone, L-glutamine, pyridoxine hydrochloride	The combination of TGF-β1 and ITS stimulated cell growth and synthesis of proteins and proteoglycans by human ADSCs.
2004 [69]	DMEM, FBS, ITS, ascorbate 2-phosphate, dexamethasone, TGFβ-1	Chondrogenic media containing TGF-β1 significantly increased protein and proteoglycan synthesis and DNA, sulfated glycosaminoglycans, and hydroxyproline content of engineered constructs.
2004 [70]	DMEM, FBS, transferrin, ITS, ascorbate 2-phosphate, dexamethasone, TGFβ-1	Chondrogenic media enabled processed lipoaspirate cells to form nodules within 48 h of induction and expressed the cartilaginous markers collagen type II, chondroitin-4-sulfate and keratan sulfate.
2006 [71]	HAMS-F12, DMEM, ITS, ascorbate 2-phosphate, dexamethasone and TGFβ-1	By day 14 ADSCs in chondrogenic media on elastin hydrogels exhibited formation of collagen and sulfated glycosaminoglycan.
2006 [72]	DMEM, FBS, ITS, ascorbate 2-phosphate, BMP-6	BMP-6 up-regulated aggrecan and collagen expression showing BMP-6 is an inducer of chondrogenesis in ADSCs.
2006 [73] *	DMEM, FBS, ITS, ascorbate 2-phosphate, BMP-2	ADSCs induced by rhBMP-2 were transplanted into nude mice and formed cartilage lacuna at week 8.
2007 [74]	DMEM, ITS, ascorbate 2-phosphate, sodium pyruvate, pyridoxine hydrochloride, L-glutamine, dexmethasone, TGF-β1, BMP-2	BMP-2 and TGF-β1 induced a chondrogenic phenotype in ADSC.
2009 [75]	HAMS-F12, DMEM, ITS, ascorbate 2-phosphate, thyroxine, pyruvate, dexamethasone, TGFβ-2, BMP-2,6,7	At 4 weeks, glycosaminoglycan assays, RT-PCR, and histology demonstrated the combination of 5 ng/mL of TGFβ-2 and 100 ng/mL of BMP-7 most effectively induced chondrogenesis of ADSCs.

Key; *TGFβ*-*1* transforming growth factor-β1, *BMP* bone morphogentic protein, *DMEM* dulbecco modified eagle medium, *FBS* fetal bovine serum, *ADSCs* adipose derived stem cells, *ITS* insulin-transferrin-selenium, *BSA* bovine serum albumin, *RT*-*PCR* real-time reverse-transcription PCR

Several synthetic scaffolds have been utilised for the chondrogenic differentiation of ADSCs. Cui et al. found that PGA scaffolds supported chondrogenesis of ADSCs to repair full thickness articular cartilage defects (8 mm in diameter, deep to subchondral bone) in femur trochlea at 3 months [78]. Poly-lactide-co-glycolide (PLGA) scaffolds were also found to support the differentiation of ADSCs for 3 weeks in vitro in media supplemented with TGF-β1 [79].

Few studies have compared differentiated and undifferentiated ADSCs for their chondrogenic potential. Rabbit osteochondral defects were treated with predifferentiated and undifferentiated ADSCs on gelatin hydrogels. Pre-differentiated ADSCs showed the highest level of cartilage formation by histological examination. Predifferentiated ADSCs were further compared to undifferentiated ADSCs after being implanted into nude mice on poly(3-hydroxybutrate-co-3-hydroxyvalerate) (PHBV) scaffolds for 16 weeks [80]. Differentiated ADSCs were found to show stronger chondrocytes-specific histochemical staining and stronger compression moduli [80]. Alginate gels also supported cartilage formation when ADSCs were pre-differentiated when subcutaneously implanted into nude mice after 20 weeks but no cartilage like tissue formation was found using undifferentiated ADSCs [81].

Despite extensive research into the chondrogenic potential of ADSCs only one in vivo study has confirmed the promising application of ADSCs for craniofacial applications. Bahrini et al. recently illustrated that ADSCs may be a novel candidate for the repair of auricular cartilage injuries in vivo. ADSCs from rabbit adipose tissue was injected into the midportion of a surgically created rabbit ear auricle cartilage defect. After 6 months mature cartilaginous plates completely filled the defect in the native cartilage [82].

It is clear that the biochemical environment, including the growth factors, hormones and specific laboratory cell culture conditions required to chondrogenic differentiate ADSCs is still being determined. Hence, further exploration into optimising chondrocyte culture conditions is required before large amount of animal studies or clinical studies are performed.

### Use of ADSCs to Generate Adipose

**Fig. 3:**
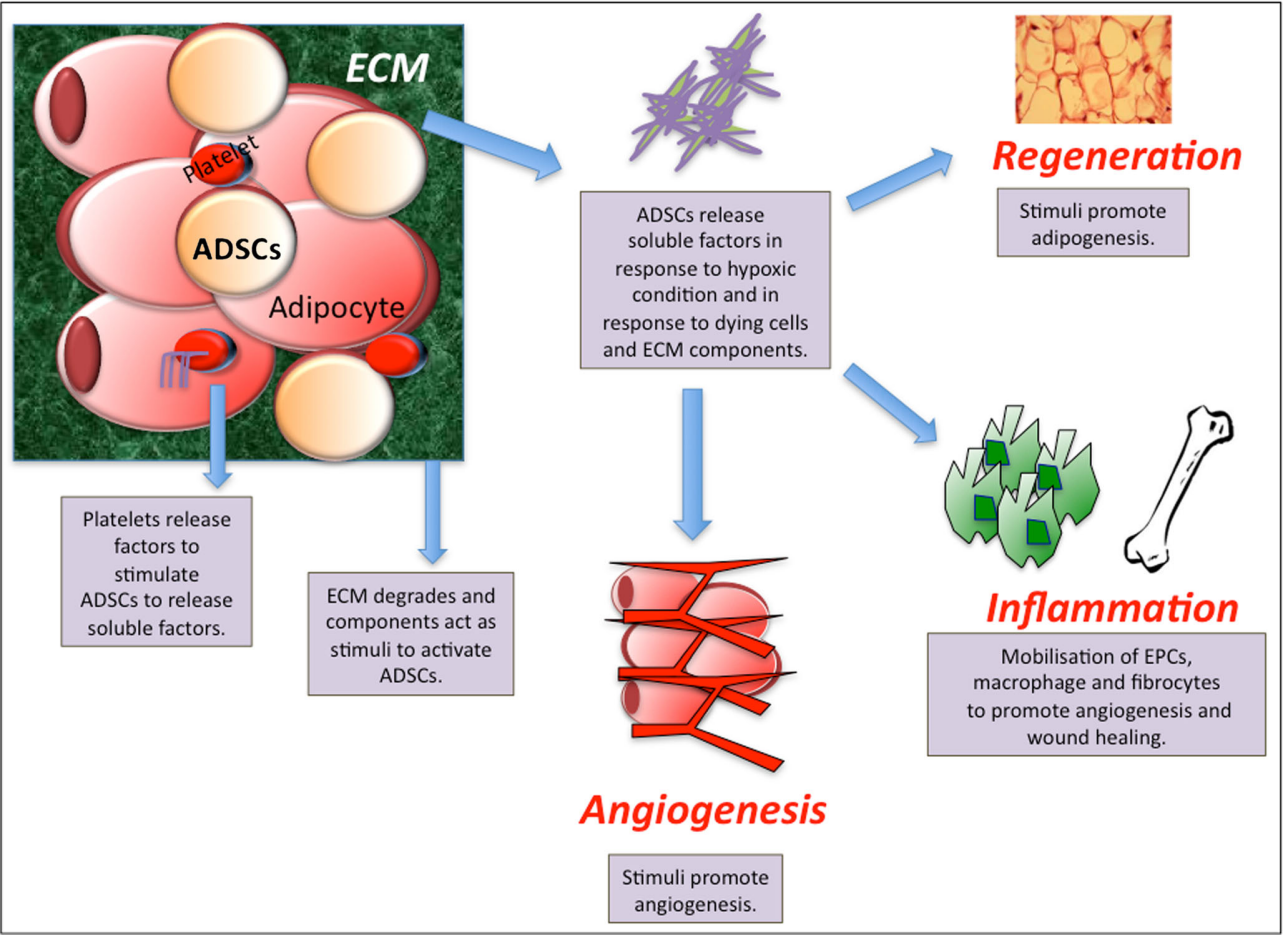
Proposed action of adipose derived stem cells (ADSCs) in enhancing survival of fat grafts. Factors released from the extracellular matrix and platelets stimulate ADSCs to secrete angiogenic factors, which stimulate angiogenesis, adipogenesis and wound healing (Adapted from Regenerative Medicine. (2011) 6(6s), 33-41 with permission of Future Medicine Ltd). ECM; Extracellular Matrix, ADSCs: Adipose derived stem cells, EPCs; Endothelial progenitor cells

Soft tissue defects range from a small to major subcutaneous tissue loss on the face from congenial, trauma or inflammatory conditions. Neuber et al. was the first to publish the use of autologous fat transplantation in 1893 for the use of facial scars [83]. Despite adipose tissue being a quick, safe and reliable method for restoring volume and utilised for over 100 years, little has be done to improve the clinical performance of fat grafts. Autologous fat grafts are associated with many difficulties including donor site morbidity, uncertain viability and behaviour of the grafted fat and a low rate of graft survival [84]. The loss of tissue has also been shown to be replaced by the conversion of the graft to fibrous tissue, and sometimes includes the formation of cysts [84]. Recently, ADSCs have been thought to overcome these limitations due to their significant potential for angiogenesis and adipogenesis [85]. When fat is grafted into the site a series of reactions have been found to occur. The bleeding at the recipient tissue activates platelets, causing the release of platelet derived growth factor (PDGF), epidermal growth factor (EGF) and TGF-β [85, 86]. The grafted fat is under severe ischaemia until a direct vascular supply is formed, causing the death of adipocytes, vascular endothelial cells but not adipose-derived stem/progenitor cells [87, 88]. The dying cells as well as the extracellular matrix (ECM) disruption cause the release of soluble factors. As the ADSCs/progenitor cells do not die they are capable of responding to these factors to release paracrine factors or simulate the mobilisation of endothelial progenitor cells (EPCs) from the bone marrow or resident progenitor cells to stimulate angiogenesis and adipogenesis [89, 90] (See Fig. 3). However, further investigation is required to confirm the action of ADSCs in grafted fat tissue to promote angiogenesis and adipogenesis as it is still unclear and not fully understood or documented [85].

Over the last decade several studies have explored the addition of stem cells to fat grafts. Zhu et al. further showed that after 6 and 9 months ADSCs enhanced fat grafts not only enhanced the longevity 2-fold compared to adipose free grafts but enhanced the expression of various growth factors including vacular endothelial growth factor-A (VEGFA) and insulin growth factor-1 (IGF-1), promoting angiogenesis and adipocyte differentiation and preventing apoptosis [91]. Moseley et al. illustrated that fat supplemented with ADSCs can improve longevity and the volume of the grafts [92].

In addition, ADSCs have also been used to enhance grafts by using the SVF to augment soft tissue survival, in a process called Cell Assisted Lipotransfer (CAL) (Fig. 1). The process begins with extracting adipose tissue using the conventional machine. The aspirate is then divided into two portions. Half of the aspirate is washed extensively with sterile phosphate-buffered saline (PBS) to remove contaminating debris and red blood cells and then treated with 0.075 % collagenase for 30 min at room temperature. The infranatant is centrifuged for 5 min at 1,200 *g* after being inactivated using FBS. The cellular pellet is then resuspended in 10 % FBS and passed through a 100-μm mesh filter to remove debris. The first portion is then also centrifuged at 1,200 *g* for 5 min and then mixed with the adipose stem cell rich aspirate for 15 min before being transplanted. Preclinical studies have illustrated the benefit of CAL [93, 94]. Aspirated fat was transplanted subcutaneously into severe combined immunodeficiency mice with CAL and without CAL. The CAL fat survived better (35 % larger on average) than non-CAL fat, and microvasculature was detected more prominently in CAL fat [93]. The study also confirmed that some of the ADSCs differentiated into vascular endothelial cells being immunopositive for Von Willebrand Factor, which could have contributed to neoangiogenesis in the acute phase of the transplantation [93]. Similarly, Lu et al. reported that fat grafts implanted in the subcutaneous tissue of 18 nude mice supplemented with ADSCs transduced with vascular endothelial growth factor (VEGF) had better survival than ADSCs free fat grafts over 6 months (74.1 ± 12.6% and 60.1 ± 17.6%, respectively) [94].

Several studies have shown that ADSCs can be delivered to the specific site via scaffolds. ADSCs are capable of attaching to synthetic and natural scaffolds can undergo proliferation, differentiation and angiogenesis. Venugopai et al. illustrated that ADSCs grown on biphasic calcium phosphate allowed the formation of fat when implanted in the rat dorsum muscle after 3 weeks [95]. PLGA adipocyte grafts have shown to maintain a phenotype after 56 days with positive confocal microscopy showed associated LipidTOX Deep Red neutral lipid staining. The PLGA scaffolds were further encapsulated within the alginate/chitosan hydrogel capsules and showed subcutaneous tissue over 28 days [96].

**Fig. 4:**
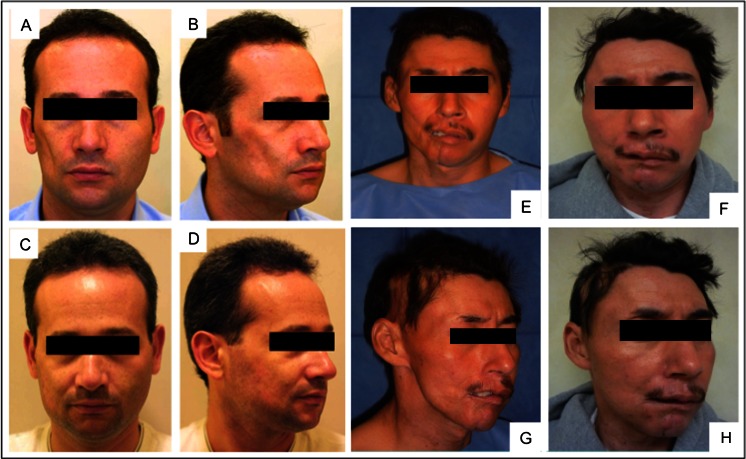
Left: Clinical views of cell assisted liposuction (CAL) for Grade 4 lipoatrophy. (A, B) Preoperative views of the patient diagnosed with Parry-Romberg syndrome (PRS). (C, D) CAL (110 mL) was performed to correct the facial defect, which improved and the facial contour was maintained at 13-month follow-up. The cheek is soft and natural appearing with no visible scars. Reprinted from Yoshimura K, Sato K, Aoi N et al. (2008). Cell-assisted lipotransfer for facial lipoatrophy: efficacy of clinical use of adipose-derived stem cells. Dermatol Surg, 34, 1178–85, copyright (2014), with permission of John Wiley & Sons, Inc. Right: A 35-year-old male patient diagnosed with Parry-Romberg syndrome (PRS). (E,G) Preoperative view. (F, H) Postoperative view 12 months after lipoinjection enriched with adipose derived stem cells. Taken with permission from Castro-Govea Y, De La Garza-Pineda O, Lara-Arias J et al. (2012). Cell-assisted lipotransfer for the treatment of parry-romberg syndrome. Arch Plast Surg, 39, 659–62

Few clinical studies have illustrated the clinical application of ADSCs for clinical use in regeneration of facial fat tissue [84, 97–102]. Yoshimura et al. in 2008 was the first to illustrate the effectiveness of CAL for facial augmentation, in patients with facial lipoatrophy [84]. Lee et al. in 2012 found CAL in 9 patients with facial augmentation gave better satisfaction and photographic evidence of increased volume than those without SVF cells (Fig. 4) [97].

Progressive facial hemiatrophy, also known as Parry-Romberg Syndrome, is a gradual loss of the subcutaneous tissue on one side of the face that creates craniofacial asymmetry (Fig. 4) [98]. A single patient underwent CAL after a 5-year history of right facial hemiatrophy. At 12 months follow up there was better volume and symmetry of the frontotemporal region and malar prominence and cheek [98]. A similar case report illustrated that ADSCs could be used for Linear scleroderma “en coup de sabre”, characterized by atrophy and furrowing of the skin of the front parietal region above the level of the eyebrow [99].

## Outlook for Adipose Stem Cell for Cranial Facial Surgery

Literature reports of ADSCs for cranial factor application have rapidly increased over the last 5 years. Though preclinical data is encouraging, largely level 4 and 5 clinical evidence with lack of power is insignificant to affect tailor clinical practice (Table 3). There are considerable hurdles, which remain to bring ADSCs into large-scale engineering for craniofacial surgery.

**Table 3:** Review of all the clinical studies that have utilised adipose derived stem cells (ADSCs) for cranial facial surgery over the last 10 years

Tissue Replaced	Year and Ref	Study Design	Methods	Outcome	Authors Conclusions
Fat	2008 [84]	3 patients underwent conventional lipoinjection (non-CAL), while 3 patients underwent CAL for facial augmentation.	1. Adipose portion of lipoaspirate digested with 0.075 % collagenase for 30 mins on a shaker at 37 °C.	CAL had a better clinical improvement score than non-CAL to 13 months follow up, although not significant (*p* = 0.11).	Longer follow up required. Safe and effective treatment.
			2. Mature adipocytes separated from the SVF containing ADSCs by centrifugation (800 g, 10 mins).		
			3. The fluid portion was centrifuged (800 g, 5 mins), and the pellets were resuspended in hypotonic water to lyse erythrocytes.		
			4. During the processing period, the other half of lipoaspirates was harvested as graft material.		
			5. The adipose portion of liposuction aspirates was centrifuged at 700 g for 3 mins without washing. In the non-CAL group, centrifuged fat was injected without SVF supplementation.		
			6. In the CAL group, the fresh SVF isolated from both the adipose and the fluid portion was added to the graft material and then put into injection syringe.		
Fat	2012 [97]	CAL against traditional soft tissue grafting in 9 patients undergoing facial augmentation with follow-up for 12 weeks.	Similar technique to 2008 [84]	Volume and patient satisfaction was significantly greater for CAL assisted facial augmentation.	No significant adverse effects.
Fat	2012 [98]	5-year history of progressive right facial hemiatrophy, who underwent facial volumetric restoration using CAL.	Same technique as 2008 [84]	At 12 months better volume and symmetry of the frontotemporal region and malar prominence and cheek.	CAL has showed promising results in the long term by decreasing the rate of fat reabsorption.
Fat	2012 [99]	19 year old with dermatoses and contour deformities on her forehead. Total of 10 × 10^7^ cell suspension in a 5-ml agitated with the 180-mL fat graft.	1. Lipoaspirate processed with pure graft system to create a pure fat graft.	Good patient satisfaction and cosmetic effect at 1 year	Prevented multiple surgeries for the patient.
			2. Graft divided into 2 parts, 180 ml spared for reconstruction and 360 ml introduced into the Celution system and processed for 2 h 25 mins.		
			3. 10 x10^7^ cell suspension in 5-mL syringe then used.		
Fat	2012 [100]	10 patients with Parry-Romberg disease. 5 received ASC and microfat grafts and 5 received microfat grafts only. Follow up 15 months	1. Extracted ADSCs isolated similarly to Yoshimura et al. 2008.	Resorption in this ADSC group was 20.59 % compared to fat only group of 46.81 %.	A microfat graft with simultaneous ADSC injection may be used to treat Parry-Romberg disease without the need for microvascular free flap transfer.
			2. Cell seeded into a culture flask and cultured overnight.		
			3. On day 14, patients were injected with secondary fat grafts and test patients simultaneously received 1 × 10^7^ ADSCs.		
Fat	2013 [101]	14 patients with craniofacial microsomia were grafted either with supplementation of ADSCs or without supplementation ADSCs.	Similar technique to 2008 [84]	Surviving fat volume at 6 months was 88 % for the experimental group and 54 % for the control group (*p* = 0.003).	Isolation and supplementation of ADSCs is effective, safe, and superior to conventional lipoinjection for facial recontouring in craniofacial microsomia.
Fat	2013 [102]	38 women who underwent fat transplantation with SVF (*n* = 26) or fat grafting alone (*n* = 12).	Similar technique to 2008 [84]	No complications were evidenced during follow-up. Fat survival was higher with SVF (64.8 ± 10.2 %) than fat grafting alone (46.4 ± 9.3 %) (*p* < 0.01).	Supplementing fat grafts with SVF for cosmetic facial contouring can improve the survival of fat grafts over fat grafting alone.
Bone	2011 [54]	4 patients with calvarial defects received autologous ADSCs seeded in bTCP granules. For 2 patients, a bilaminate technique with resorbable mesh was used.	1. ADSCs were grown under Good Manufacturing Practice for 22 days.	3 months no complications and CT scans ossification was similar to native bone.	The combination of scaffold material such as bTCP and autologous ADSCs constitutes a promising model for reconstruction of human large cranial defects.
			2. 15 x10^6^ cells of passage 3 and 4 were subsequently combined with 60 mL of bTCP granules for 48 h before the operation.		
Bone	2004 [55]	Calvarial defect of 7-year old using fibrin glue and ADSCs from the iliac bone.	1. ADSCs from the gluteal region during harvesting of the bone graft from iliac crest.	No complications and union at 3 months.	Further studies, both in vitro and in vivo, are needed to turn this first case into a reproducible and reliable treatment regimen in craniofacial bone reconstruction.
			2. Processing and isolation for 2 h.		
			3.10 ml of the prepared solution of ADSCs was evenly applied to the cancellous bone grafts.		
			4.To keep the cells in place, 8 ml of autologous (obtained preoperatively by plasmapheresis and cryoprecipitation) fibrin glue was applied using a spray adapter.		
Bone	2009 [61]	Maxillary reconstruction following hemimaxillectomy due to a large keratocyst. ADSCs were expanded for 14 days prior to be seeded on a titanium cage with bTCP.	1. First operation was used to extract the ADSCs, which was then expanded for 14 days.	After 8 months, the flap had developed mature bone structures and was placed in the area without complication.	This is the first clinical case where ectopic bone was produced using autologous ADSCs in microvascular reconstruction surgery.
			2. Prior to combining the cells with beta-TCP, the beta-TCP was incubated for 48 h in basal medium containing 12 mg rhBMP-2		
			3. Following the incubation, the media containing rhBMP-2 was discarded. Subsequently, to allow cell attachment, approximately 13 x10^6^ cells were combined with 60 ml of bTCP granules 48 h prior to the operation.		
			4. In the second operation a titanium cage filled with ADSCs and bTCP was inserted into the left rectus abdominis muscle.		
			5. The rectus abdominis free flap was then raised 9 mths later to open the cage, disconnect the vessels and then the flap was placed in the maxillary defect.		
Bone	2010 [60]	14-year-old adolescent boy with Treacher Collins syndrome whose bilateral orbitozygomatic defects were treated with engineered bone made from a combination of human bone allograft, ADSCs, BMP-2, and periosteal grafts.	1. 28 mm of fresh lipoaspirate from the abdomen was pipetted onto the bone allograft	The reconstruction remained stable during a 6-month follow-up, biopsy of the engineered bone showed health, lamellar bone.	The combination of ADSCs, BMP-2, bone allograft, and periosteum may provide an alternative method to both osteocutaneous free flaps and large structural allografts with less morbidity and improved long-term results.
			2. After this, each construct was covered with recombinant human BMP-2 on a collagen sponge.		
			3.Lastly, periosteal grafts from the patient’s left femur were sewn into position over the bilateral bony constructs.		
Bone	2012 [58]	All patients with jaw defects were reconstructed with ADSCs, resorbable scaffolds, and growth factor as required. Vascularized soft tissue beds were prepared for ectopic bone formation and later microvascular translocation as indicated.	Same technique as 2009 [61]	23 ADSCs seeded resorbable scaffolds combined with rhBMP-2 were successfully implanted to reconstruct jaws except for three failures (one infection and two cases of inadequate bone formation).	ADSC-aided reconstruction of large defects remains challenging as it takes longer and has a higher cost than the conventional standard immediate reconstruction but results are encouraging.
Bone	2013 [59]	10 cm anterior mandibular ameloblastoma resected and repaired using β-TCP granules, recombinant BMP-2, and Good Manufacturing ADSCs.	Similar technique to 2009 [61]	After 10 months dental implants could be implanted and prosthodontic rehabilitation was completed.	ADSCs in combination with β-TCP and BMP-2 good option for mandibular defects without the need for ectopic bone formation and allowing rehabilitation with dental implants.

Key; *ADSCs* adipose derived stem cells, *bTCP* beta tricalcium phosphate, *CAL* cell assisted lipotransfer, *SVF* stromal vascular fraction

There are many unanswered questions that limit the clinical translation of ADSCs for craniofacial application relating to the isolation and processing of ADSCs. Firstly, whether optimal tissue formation is produced using cultured or uncultured ADSCs need to be determined. Discovery of specific markers for ADSCs is also vital as this will allow rapid purification of ADSCs, which may allow immediate use without cell culture. The application of ADSCs currently remains costly and time consuming. Reliable fast and efficient protocols for expansion and differentiation of ADSCs into bone, cartilage and adipose is also required before large clinical trials are to be carried out. Standardized harvesting, processing and differentiation protocols would also allow clinical studies to be able to effectively compare clinical data. The number of cells that need to be harvested for effective implantation is not clear from the literature when direct application of ADSCS is utilised as for cell assisted lipotransfer.

ADSCs capacity to heal cranial defects has been investigated to the greatest depth in the literature to date, both in preclinical and clinical studies. Several in vivo animal studies have highlighted ADSCs can heal bone defects to the calvarial and mandible, with few supporting clinical studies. However, it is still not clear whether scaffolds, growth factors are required for optimal osteogenesis over a long period of time. Furthermore, the specific scaffold material to support bone growth is unknown and the importance of supplemental growth factor in particular BMPs needs to be explored. Few studies have illustrated the capacity of in vivo cartilage formation using ADSCs for cranial defects [82]. Further study into differentiation protocols of ADSCs into cartilage, scaffold biocompatibility and suitability will further develop the potential for ADSCs to heal cranial chondrogenic defects [66]. ADSCs capacity to heal adipose cranial defects has attracted considerable research interest. Clinical studies have illustrated the potential of ADSCs to enhance fat loss due to facial hemiatrophy, Parry Romberg disease and natural soft facial defects. However, due the small numbers, variability in dose of ADSCs used and processing of ADSCS, variability in patient populations in these studies, further in vitro and animal studies are required to expand ADSCs clinical applications. Greater understanding of the ADSCs mechanisms to promote angiogenesis and angiogenesis will enable researchers to control the amount of soft tissue formation. The paracrine factors, migratory stimuli and differentiation potential of ADSCs to enhance angiogenesis and soft tissue formation needs to be analysed at a greater depth.

## Conclusion

Preclinical data and few clinical studies have highlighted the potential of ADSCs for generation of required bone, cartilage and fat tissue for craniofacial surgery. A greater understanding into the mechanisms that control ADSC differentiation, immunodulatory functions and angiogenesis capacity is vital to expand the use of ADSCS in craniofacial surgery. Furthermore the optimal harvesting and processing techniques of ADSCs for cultured and uncultured ADSCs needs to be determined. This acquired knowledge will allow surgeons and researchers in the future to control ADSCs in expansion or through direct implantation providing optimal tissue restoration for craniofacial applications. Currently we are working in extraction of ADSCs in frametime of an operation of 2–3 h, which may reduce the regulatory hurdle in the future.
